# Oncogenic Functions of the Cancer-Testis Antigen SSX on the Proliferation, Survival, and Signaling Pathways of Cancer Cells

**DOI:** 10.1371/journal.pone.0095136

**Published:** 2014-04-30

**Authors:** Padraig D’Arcy, Wessen Maruwge, Barry Wolahan, Limin Ma, Bertha Brodin

**Affiliations:** Department of Oncology-Pathology, Cancer Center Karolinska, Stockholm, Sweden; Duke University Medical Center, United States of America

## Abstract

SSX is a transcription factor with elusive oncogenic functions expressed in a variety of human tumors of epithelial and mesenchymal origin. It has raised substantial interest as a target for cancer therapy since it elicits humoral responses and displays restricted expression to cancer, spermatogonia and mesenchymal stem cells. Here, we investigated the oncogenic properties of SSX by employing a RNA interference to knock-down the endogenous expression of SSX in melanoma and osteosarcoma cell lines. Depletion of SSX expression resulted in reduced proliferation with cells accumulating in the G1 phase of the cell cycle. We found that the growth promoting and survival properties of SSX are mediated in part though modulation of MAPK/Erk and Wnt signaling pathways, since SSX silencing inhibited Erk-mediated signaling and transcription of cMYC and Akt-1. We also found that SSX forms a transient complex with β-catenin at the G1-S phase boundary resulting in the altered expression of β-catenin target genes such as E-cadherin, snail-2 and vimentin, involved in epithelial-mesenchymal transitions. Importantly the silencing of SSX expression in *in vivo* significantly impaired the growth of melanoma tumor xenografts. Tumor biopsies from SSX silenced tumors displayed reduced cyclin A staining, indicative of low proliferation and predominantly cycloplasmic β-catenin compared to SSX expressing tumors. The present study demonstrates a previously unknown function of SSX, that as an oncogene and as a tumor target for the development of novel anti-cancer drugs.

## Introduction

*SSX* was initially identified as part of the *SS18/SSX* fusion gene in synovial sarcoma [1] and as the melanoma associated tumor antigen HOM-Mel40 [2]. It consists of a family of nine, highly homologous genes organized in clusters on the X chromosome with products classified as cancer-testis antigens based on their restricted expression in tumors and testis. In normal cells, SSX expression has been found in spermatogonia [3], [4], mesenchymal stem cells [5]. The expression of SSX family members in tumors has been extensively investigated, and it has been shown that SSX1, SSX2, SSX4 and SSX5 are expressed independently or simultaneously often displaying widespread, scattered or focal expression patterns in tumors of epithelial, hematopoietic, neural and mesenchymal origin [3], [6]–[8].

The protein is rich in charged amino acids [9], and contains two so called repressor domains that represses transcription *in vitro*; a Krüppel associated box localized at the N terminus, and a stronger repressor domain (RD) at the C-terminus [10], [11]. In cells, SSX has a granular expression pattern and localizes in the nucleus and the cytoplasm [5], [12]. Direct interaction of SSX with DNA has not been demonstrated, it is therefore assumed that SSX repress transcription by forming complexes with DNA binding proteins. In support of this model both SSX and SS18/SSX fusion gene product have been shown to interact with members of the polycomb repressor complex Bmi-1 and Ring 1 [13], and core histones [14] suggesting that SSX may control the expression of genes regulating cell differentiation. Other proteins that interact with SSX are the Ras-like GTPase binding protein RAB3IP, the nuclear protein SSX2IP [15], and the LIM homeobox protein LHX4 [16].

Since its discovery as a cancer-testis antigen, the immunotherapeutic targeting of SSX has raised great interest as an anti-cancer strategy due to its immunogenicity [2], [3], restricted tumor expression, and correlation between SSX expression and disease progression [8], [17], [18]. T-cell cytotoxic responses have been generated *in vitro* against SSX epitopes [19]–[21], however, the validation of SSX as a therapeutic target *in vivo* has not been reported. In the present investigation we have evaluated the role of SSX in mediating cell growth and survival of cancer cells, in *vitro* and *in vivo*, and identified growth signaling pathways SSX expression.

## Results

### SSX2 is Preferentially Expressed in Metastatic Melanoma Lesions and Derived Cell Lines but not in Normal Cells

**Figure 1:**
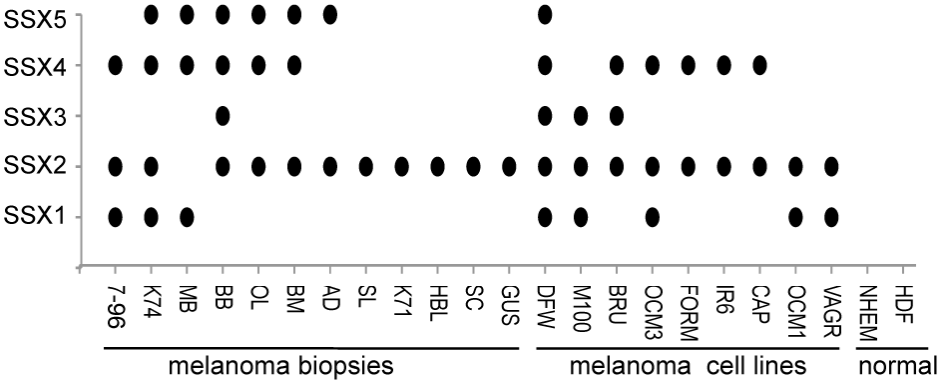
SSX2 is frequently expressed in melanoma lesions and derived cell lines but not in normal cells. SSX expression was analyzed by a nested RT-PCR method previously described using primers recognizing SSX1 to SSX 9 cDNA. Fresh biopsies were obtained from metastatic lesions of melanoma patients. The DFW melanoma cell line expressing high levels of SSX1 to SSX5 was used for RNAi studies. NHEM: normal human epithelial melanocytes, HDF: human diploid fibroblasts.

We investigated the expression of SSX1 to SSX5 in 12 metastatic melanoma lesions, 9 early passaged melanoma cell lines, normal human epithelial melanocytes (NHEM) and human diploid fibroblasts (HDF) using a sequencing validated RT-PCR method previously described [5]. Similar to other published studies we found that several SSX transcripts were simultaneously expressed in all melanomas and melanoma cell lines examined. Interestingly SSX2 was detected in almost all melanoma tissues and derived cell lines (95%) compared to SSX4 (57%), SSX1 (38%), SSX5 (33%) and SSX3 (19%) expression. None of the SSX members were detected in normal human epithelial melanocytes (NHEM) or in normal human diploid fibroblasts (HDF) (Figure 1). The sequence of the primers used for detection of SSX-1 to SSX-9 and the expression of SSX in osteosarcomas cell lines including the line used in this study SAOS-2 is shown in Figure S1.

### The Conditional Silencing of SSX Inhibits Tumor Cell Proliferation by Blocking Entry of Tumor Cells into S-phase

**Figure 2:**
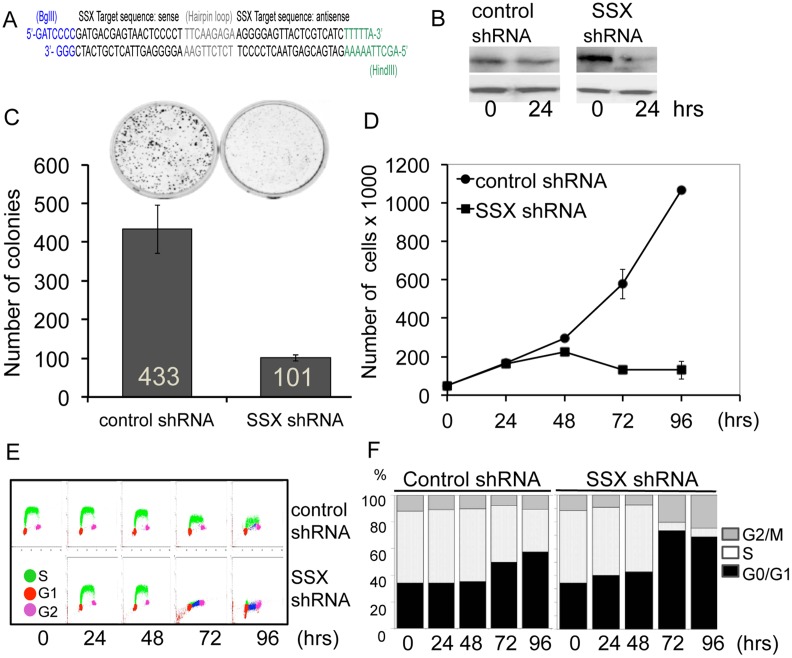
The conditional knock-down of SSX inhibits the proliferation, survival and cell cycle progression of the melanoma cell line DFW in vitro. A) Graphic representation of the shRNA sequence (complementary to SSX1 to SSX9) ligated into shRNA vectors for stable and doxycycline regulated shRNA expression (see material and methods). B) Western blot showing SSX expression in control-shRNA and SSX-shRNA transfected cells 24 hours after doxycycline addition to the culture medium. C) Cell colony quantification in control and SSX-shRNA transfected DFW cells grown in the presence of doxycycline for 8 days. D) Cell proliferation curves determined by counting the number of alive cells in control and SSX silence cultures using trypan blue staining. E) S-phase cell cycle progression determined by BrdU incorporation in a fluorescence activated cell sorter (FACS). F) Percentage of cells at G1, S and G2 phases of the cell cycle in control and SSX shRNA knocked down DFW cells over 96 hours period.

To get an insight into the function of SSX in tumor cells, we silenced SSX in the melanoma cell line DFW that expresses SSX1 to SSX5 (Figure 1) using plasmids for both stable and doxycycline conditional expression of shRNA molecules targeting SSX1–9 transcripts (Figure 2A and Figure S2) as described in material and methods.

**Figure 3:**
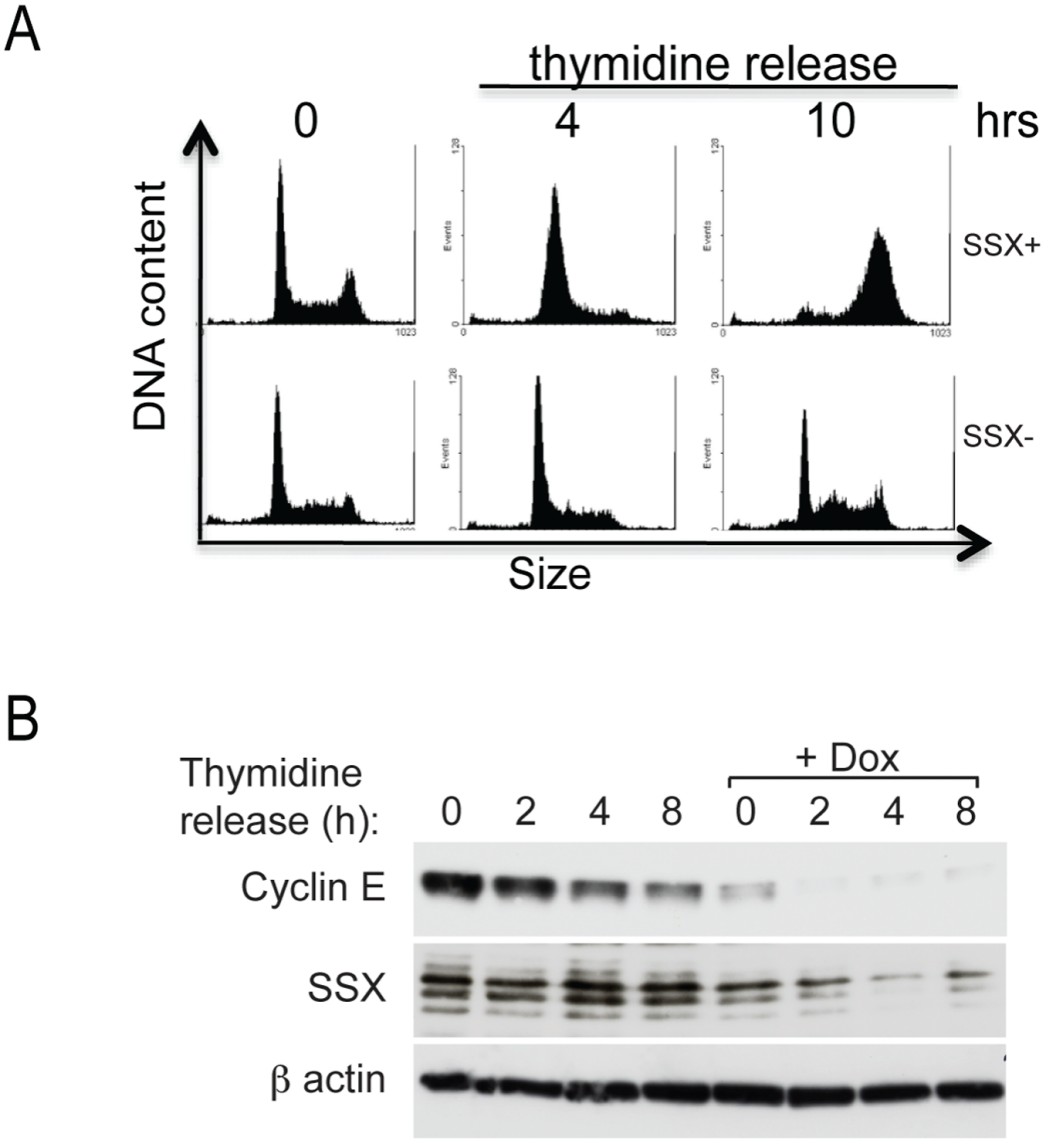
Loss of SSX expression arrest tumor cells in G1/S phase and abrogates -cyclin E expression. Control (SSX+) and silenced (SSX−) DFW melanoma cells were synchronized in G1/S phase by double thymidine blockade and released in normal medium as described in material and methods. A) Entry of the cells to the S phase (S) was analyzed by quantifying DNA content using propidium iodide (PI) incorporation and a fluorescent activated cell sorter FACS. B) Western blot showing time dependent expression of SSX and cyclin E in control and SSX silenced (+Dox) DFW cells. Cells were syncronized in G1/S (0 h), released into normal medium containing FBS and harvested at the indicated time points.

Conditional silencing of SSX was induced by the addition of doxycycline into the medium and resulted in a significant decrease of SSX protein after 24 hours (Figure 2B). Cell viability counts using trypan blue exclusion cells showed the presence of viable but non-proliferating cells in the presence of doxycycline indicating that SSX expression is necessary for cell growth (Figure 2D). To investigate the validity of this observation we also performed siRNA knockdown of SSX expression in two additional osteosarcoma cell lines, U2-OS and Saos-2, using RNAi molecules targeting SSX1–9, or specific for SSX1 and SSX2. Similarly to the previous results SSX depletion resulted in reduced proliferation compared to control siRNA indicating that the phenotype observed is a bona fide effect of SSX knockdown and not due to off-target effects (Figure S2). In clonogenic assays, the silencing of SSX in DFW tumor cells resulted in an impaired colony formation as observed by a four-fold decrease in the number of colonies in cells grown in the presence of doxycycline for 14 days compared to controls (Figure 2C). Cell cycle analysis of DFW cells in following the addition of doxycycline showed that the cells failed to enter the S-phase of the cell cycle (Figure 2D). The percentage of cells in the S-phase dropped from 50% (at 0 and 24 hrs) to 5% (at 72 and 96 hrs) along with a concomitant accumulation of cells in G1 phase, indicative of a defect in cell cycle progression (Figure 2F). To confirm this effect of SSX expression on S phase entry, we synchronized wild type (SSX+) and SSX knocked-down DFW cells in G1/S phase by double thymidine block and release into normal FBS containing medium. Control (SSX+) DFW cells rapidly progressed from G1 into S-phase 4 to 10 h after release from thymidine block. In contrast SSX-knockdown cells could not traverse the G1/S phase boundary, with cells remaining arrested in the G1 phase (Figure 3A). Consistent with this observations, levels of the cyclin E, the regulating component of the cyclin E-CDK2 complex and a key regulator of G1 and S-phase progression, was decreased in parallel to the down regulation of SSX. (Figure. 3B).

### SSX Mediates Tumor Cell Growth through Mapk/Erk Signaling Pathway

The finding that SSX sustains cell proliferation and is required for the entry of tumor cells into S-phase prompted us to investigate if this effect was associated with signaling cascades that stimulate cell proliferation and survival. We began by comparing the potential of SSX expressing and knockdown cells to activate (phosphorylate) the intracellular messengers Erk and Akt-1, following growth factor stimulation.

**Figure 4:**
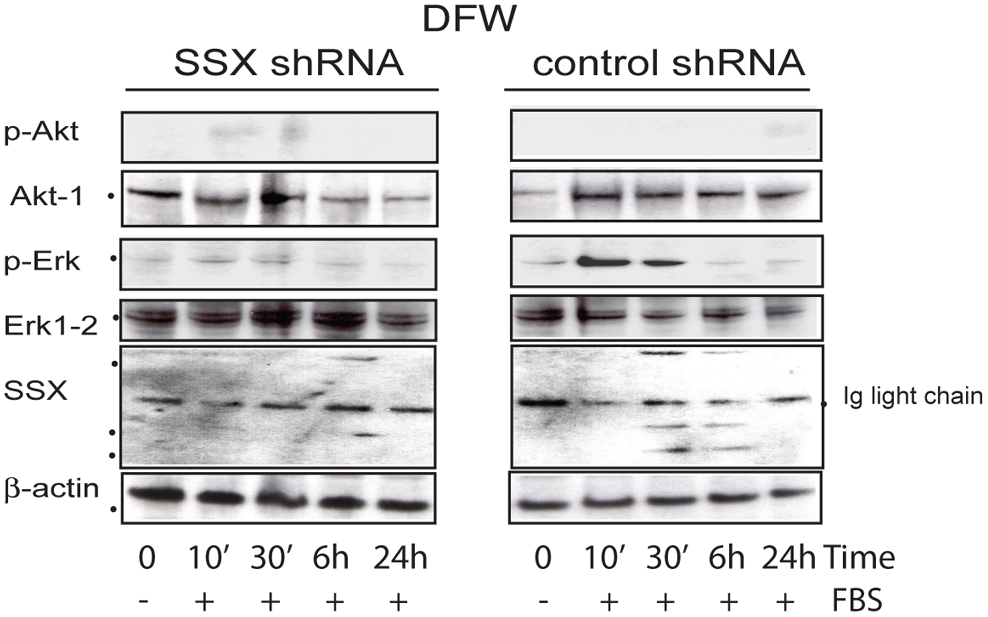
SSX is required for Erk mediated signalling. Western blot showing the expression of Erk1–2 and Akt-1 and their activated (phosphorylated) forms pAkt (Ser 473) and pErk (Thr202/Tyr204) in control shRNA and SSX-shRNA DFW cells. Cells were starved in serum free media for 36 hrs (0 h) and cell signalling was activated by the addition of serum into the media. Samples were collected at 10 and 30 minutes (10′ and 30′) and at 6 and 24 hours (6 h, 24 h) following serum stimulation. SSX expression was determined by immunoprecipitation (fl188 antibody) and western blot (N18 antibody) the later recognozing bands of approximately 22 and 14 kDa.

Loss of SSX expression was associated with decreased phosphorylation of the extracellular signal-regulated kinase (Erk) 1 and 2 but not of Akt-1 following stimulation with FBS. A decrease in the protein levels of Akt-1 was however observed in SSX silenced cells (Figure 4). Our results suggest that the effects of SSX on tumor cell proliferation are linked at least in part to modulating the activity of the MAPK/Erk signaling pathway.

### SSX Interacts with β-catenin to Regulate Transcription of EMT Associated β-catenin Genes

The 3′ region of the SSX-1, 2 and 4 genes are fused to nearly the entire SS18 gene in synovial sarcomas. Interestingly the SS18/SSX2 fusion protein forms a complex with β−catenin resulting in the activation of a T-cell factor TCF/lymphocyte enhancer factor (Lef) reporter construct when ectopically expressed in mammalian cells [22]. We therefore investigated if this interaction is conserved for the full-length SSX proteins.

**Figure 5:**
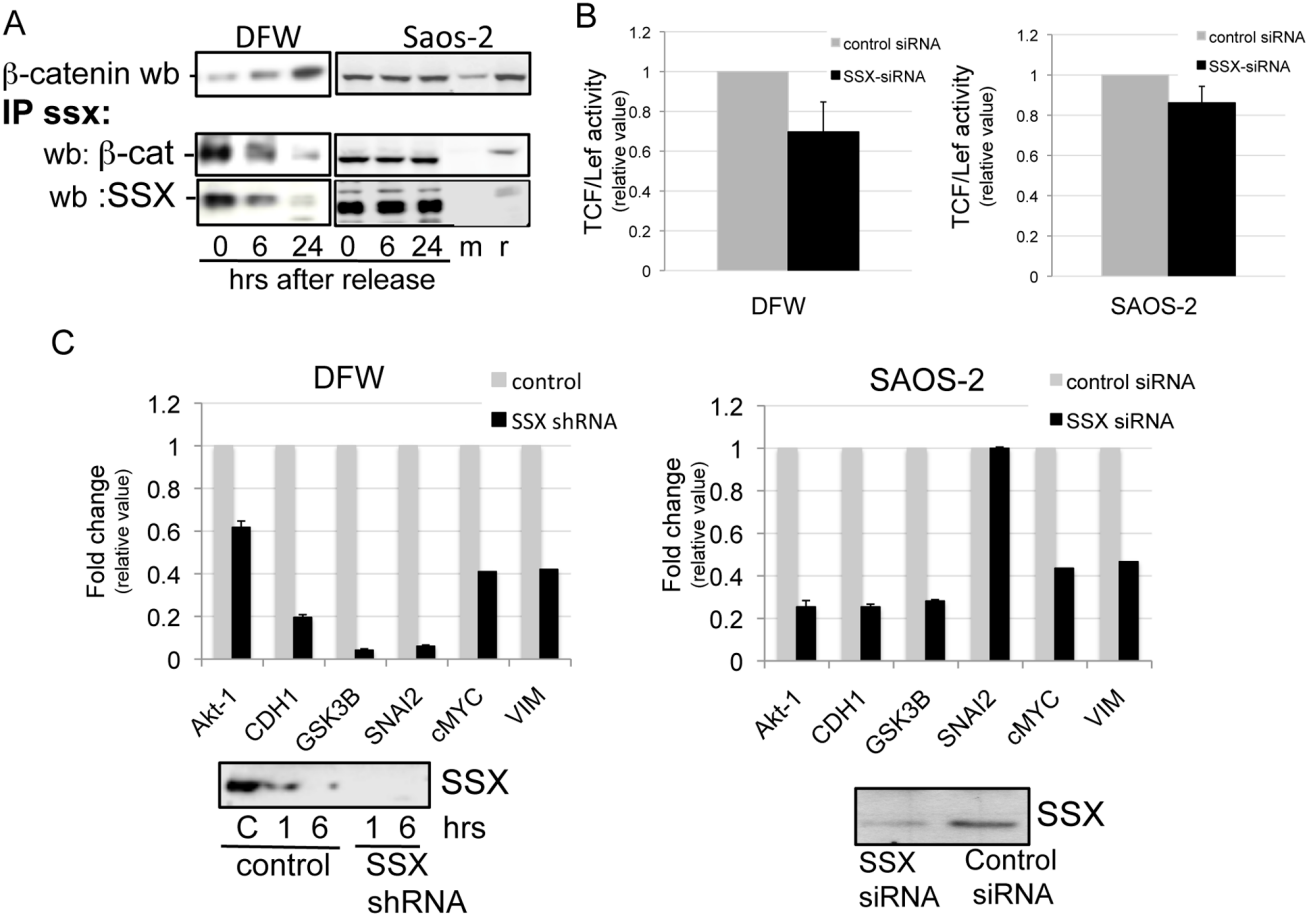
SSX interacts with β-catenin and transactivates TCF/β-catenin target genes. **A**) DFW and Saos-2 cell lines were synchronized in G1/S by double thymidine blockade as indicated in material and methods (0 hrs), and released into normal medium containing FBS for 6 and 24 hrs. SSX was immunoprecipitated from protein extracts collected at the indicated time points using the rabbit anti SSX antibody (FL188, detecting SSX1–9). An equivalent amount of protein from G1/S blocked Saos-2 cells was immunoprecipitated with an irrelevant anti mouse (m) or ant-rabbit (r) antibody. The protein complex were electrophoresed in reducing conditions and blotted ether with goat anti SSX (N18) or mouse anti β-catenin antibodies. The total input levels of β-catenin are shown in the upper gel image. B) Activity of a TCF/Lef luciferase reporter in SSX silenced and control DFW and Saos-2 cells, 48 hours after transfection of siRNA molecules (n = 5). The activity of the TCF/Lef reporter in SSX silenced cells is relative to that of control cells ( = 1). C) Gene transcription associated with SSX expression in both Saos-2 and DFW cells, determinded by PCR arrays containing 84 genes associated with epithelial to mesenchymal transition (n = 5) and confirmed by Q-RT PCR in SSX silenced and control DFW and Saos-2 cells as described in material and methods. SSX was knocked down in Saous-2 or DFW cells using siRNA molecules or shRNA vectors as indicated. Cells were collected and RNA was isolated 6 ours after siRNA transfection or 6 hours after addition of doxycycline into the medium (conditionally shRNA). The loss of SSX expression following RNAi silencing was confirmed by western blot before each Q-RT-PCR array, as shown in the figure. Fold-Change [2∧(−Delta Delta Ct)] is the normalized gene expression [2∧(−Delta Ct)] in SSX silenced cells divided by the normalized gene expression in the control (SSX+) cells. Values less than one indicate a negative or down-regulation.

Since SSX expression varies with cell cycle progression, we synchronized DFW and Saos-2 cells (time 0) at the G1/S phase boundary and released into cell the cycle for 6 and 24 hrs and performed immunoprecipitation on cell lysates with SSX antibodies and immunoblotting with β -catenin antibodies (Figure 5a). β-catenin was co-precipitated with SSX from both Saos-2 and DFW cells blocked in G1/S (time 0) as well as from cells that were released into cell cycle for 6 and 24 hrs. To ensure specificity, equal protein amounts of Saos-2 cell extracts were immunoprecipitated with irrelevant immunoglobulins from mouse (M) and rabbit (R) as controls (Figure 5A).

The reverse experiment was also performed in which cell extracts from DFW were precipitated with β-catenin antibodies and blotted with anti-SSX antibodies. Immunoprecipitation of β-catenin resulted in the co-precipitation of SSX. Of notice is that the yield of SSX obtained following β-catenin antibodies was lower than the reverse immunoprecipitation, which may be due to increased competition of β-catenin binding with other proteins (Figure S3).

In the canonical Wnt pathway, β-catenin binds to TCF/Lef to activate transcription of genes associated with cell proliferation, survival, motility and differentiation [23]. Based on this we investigated if the interaction of β-catenin with SSX impacts the transcription of TCF/β-catenin target genes. Firstly we determined the activity of a TCF/Lef-luciferase reporter in SSX-knocked-down and control (SSX+) cells. DFW and Saos-2 cells were transfected in tetraplicates with a TCF/Lef- firefly luciferase reporter and with either siRNA to SSX or control molecules. The activity of the reporter was evaluated 48 h after transfection. Interestingly we observed a decrease in reporter activity siRNA-SSX treated cells in five independent experiments (Figure 5B). We next investigated whether the SSX/β-catenin interaction was associated with changes in the transcription of endogenous β-catenin/TCF target genes. To this end we compared the transcription profiles of control and SSX knockdown using RT-PCR arrays for 84 transcripts associated with Epithelial to Mesenchyma (EMT) transitions in Saos-2 cells. We chose genes associated with EMT based on the role of β-catenin in promoting EMT and our previous report showing that SSX expression is associated with the invasive capacity of tumor cells and with expression of mesenchymal genes [5]. The results obtained in 5 independent arrays were confirmed by RT-PCR in the DFW cell line. Of 84 analyzed genes we found that the loss of SSX expression in Saos-2 and DFW was associated with reduced transcription of Akt-1, E-cadherin (CDH1), GSK3β, Snail 2 (SNAI2), c-myc (cMYC), and vimentin (VIM) (Figure 5C). With the exception of Akt-1, all these genes carry DNA binding sequences for the TCF/Lef and are therefore considered β-catenin targets. We also observed an increased collagen 3A transcription following down regulation of SSX in Saos-2 (Table S1).

### siRNA Targeting of SSX Impairs Tumor Xenograft Growth

**Figure 6:**
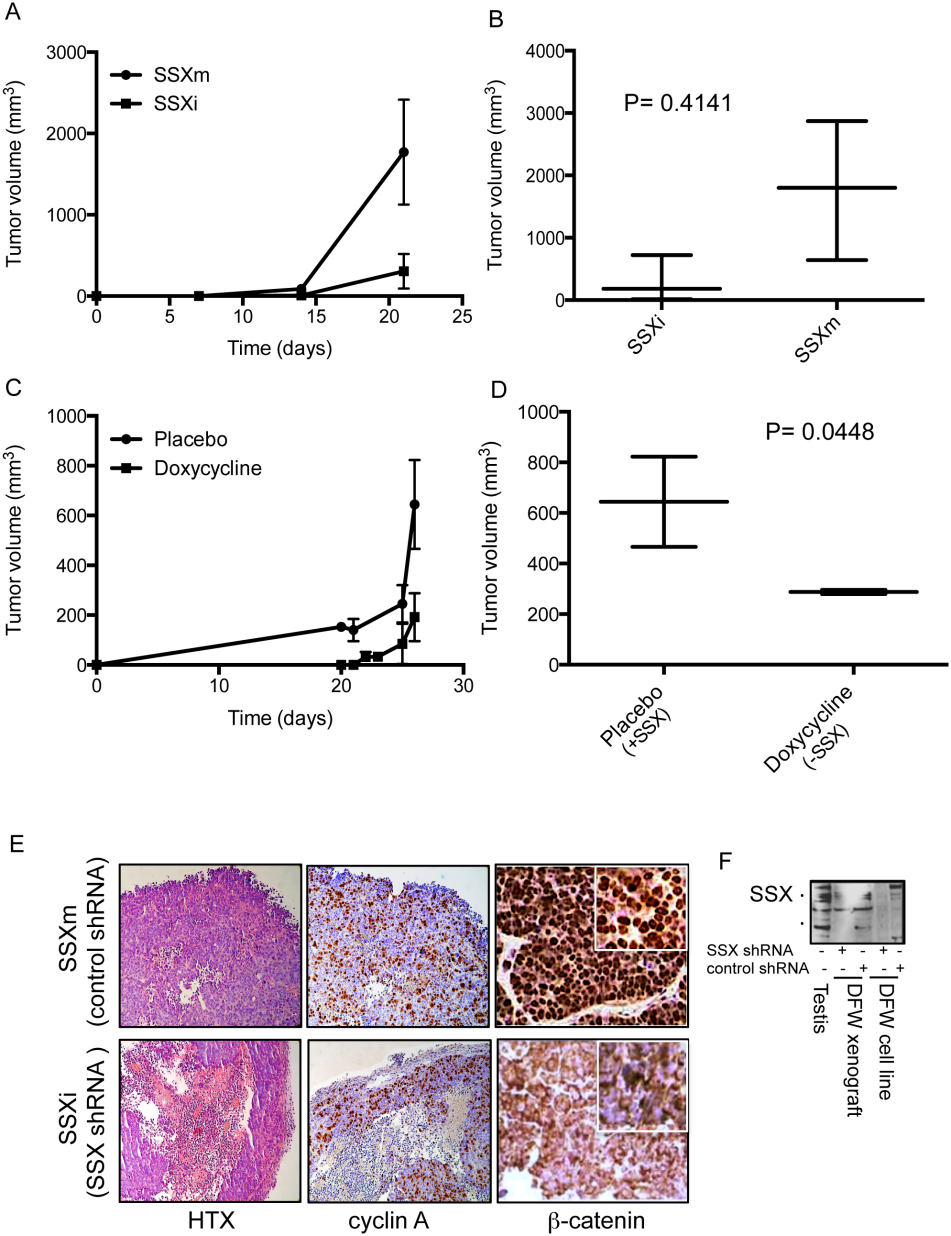
shRNA targeting of SSX in inhibit the growth of xenografts in SCID mice. A) DFW melanoma cells stably transfected with SSX-shRNA (SSXi) or with a control shRNA (SSXm) vector, expanded and xenografted in SCID mice. A) Tumor volume determined at the indicated times. B) Tumor volume at the end point of the experiment (21 days). C) Growth curves of xenografts from conditionally SSX-shRNA silenced DFW cells (SSX−) and control shRNA (SSX+) cells. Doxycycline was administered to all mice by subcutaneous insertion of a slow release pellet to mantain steady concentration of doxycycline (10 µM) for 21 days. D) Tumor volume at the end point of the experiment. E) Immunohistochemistry of the tumors visualized in the light microscope using 10x objective, inserts 63x magnification: hematoxillin (HTX), the proliferation marker (ki-67) and β-catenin (β-cat).

Having shown that SSX is required for tumor cell grow *in vitro*, we examined the growth of control and SSX silenced xenografts in mice. We subcutaneously injected SCID mice with either control-shRNA (SSXm) or SSX-shRNA (SSXi) stable transfected DFW cells (Figure 5A–B), or with DFW cells in which the shRNA expression was conditionally regulated with doxycycline (Figure 5C–D). In the conditional system, SSX knockdown was induced by subcutaneous implantation of slow release doxycycline pellets as described in material and methods. Compared to control (SSX+) tumor xenografts, SSX knockdown tumors showed impaired growth as observed by reduced tumor growth curves and by reduced tumor volume at the endpoint of the assay (Figure 6 A–D). Microscopically, SSX negative tumors displayed extensive necrosis, up to 80% and had delineated borders (Figure 6E, HTX) with local proliferating cyclin A positive cells. In contrast control tumors showed radial cell growth with abundant proliferative (cyclin A positive) areas and nuclear β-catenin (Figure 6E). Interestingly SSX knockdown tumors showed a predominantly cytoplasmic localization of β-catenin compared with control tumors, possibly indicating a role for SSX in the cellular localization of β-catenin (Figure 6E). We confirmed SSX knockdown in tumors by western blot on fresh tumor biopsies. (Figure 6F).

These results demonstrate that the down-regulation of SSX expression impairs tumor growth *in vivo* and results in altered β-catenin localization.

## Discussion

The SSX proteins are encoded by genes that are only expressed in several cancer subtypes with expression in normal tissues restricted to germ cells, trophoblasts and fetal mesenchymal stem cells. Given this restricted expression, the SSX antigens are attractive targets for tumor immunotherapy [21]. However, the function of the SSX proteins in spermatogenesis or tumorgenesis is poorly defined. SSX is expressed in distinct subpopulations of spermatogonia and in fetal mesenchymal stem cells suggesting a role for SSX in cell differentiation [4], [5]. In tumors, SSX increases invasive potential and represses E-cadherin expression, as has been shown in melanoma [5] and breast cancer cells, respectively [24]. Our results show that the expression of SSX is essential for the entry of tumor cells into S-phase of the cell cycle and, consequently, tumor cells that express SSX sustain cell proliferation and long-term survival. These functions may be associated with the ability of SSX to modulate MAPK/Erk, Akt and β-catenin signaling pathways. Consistent with a role of SSX in cell proliferation, knockdown of SSX blocked pERK activation a key component of the proliferation cascade initiated by extracellular growth factor kinases. In addition SSX knockdown also resulted in the reduced expression of Akt, a cell signaling kinase with a central role in a wide number of cellular functions including cell growth, metabolism and survival [25]. In support of this, recent reports have shown that SSX is essential for melanoma cell proliferation [26] and for the invasion capacity of breast cancer cells [24].

We found that SSX directly interacts with β-catenin in G1 arrested cells and that this interaction affects transcription of β-catenin/TCF target genes since the silencing of SSX expression was associated with the decreased activity of a TCF/Lef reporter construct and decreased transcription of β-catenin/TCF target genes such as E-cadherin, GSK3b, snail-2, vimentin and c-Myc.

β-catenin is a powerful transcription factor with a large list of target genes involved in cell proliferation, stemcellness and in epithelial to mesenchymal transitions (EMT). In a previous report we proposed a role for SSX in EMT based on our findings that in a melanoma cell line and in fetal mesenchymal stem cells, the expression of SSX was associated with a mesenchymal phenotype such as increased cell invasion capacity, decreased E-cadherin and increased matrix proteinase 2 (MMP2) expression [5]. We now demonstrate that SSX interacts with β-catenin and based on our transcription profile data propose that this interaction may induce or sustain a mesenchymal phenotype. The activity of the SSX/β-catenin complex at endogenous target sites may however be dependent on the cell lineage, time, and signaling from the microenvironment.

SSX is a current target for the development of cell-based immunovaccines. HLA A2 restricted cytotoxic T-cells (CD8+) that recognize SSX peptides have been isolated from lymph nodes from an SSX2 seropositive melanoma patient [19] and have also been generated for the immunotargeting of prostate cancer cells *in vitro* [21]. Furthermore, it has been reported that the treatment with autonomous dendritic cells primed with SSX peptides resulted in tumor remission of a synovial sarcoma patient [27]. In this report, we show that the transcriptional silencing of SSX inhibits the growth of melanoma xenografts adding further support to the significance of SSX as therapeutic target.

## Materials and Methods

### Ethics Statement

All animal work has been approved by the Swedish central board for animal studies (Centrala Försöksdjur nämnden, CFN), Stockholm North. Ethical approval numbers N399/07, N340/10 to B.B. The animal studies have been conducted according to their ethical guidelines. Collection of clinical specimens for research was done with the patient concent and approved by the Karolinska, forskningsetikkommitté Nord No 03–019. All data was analyzed anonymously and according to the principles expressed in the Declaration of Helsinki.

### Cell Lines

DFW is a HLA-A2 melanoma cell line obtained from a metastatic melanoma tumor. The line was generated at the Karolinska Institutet [28], and kindly provided by R. Kiessling. SAOS-2 (SArcoma OSteogenic) is a cell line generated from a primary osteosarcoma [29] and U2OS is an osteosarcoma cell line, both cell lines were provided by M. Fritsche, Universitát Freiburg, Germany. All cell lines detect high levels od SSX1 to SSX5, determined using RT-PCR (Figure 1 and Figure S1). The cells were grown as monolayer cultures in RPMI medium (Sigma) supplemented with 10% tetracycline-free FBS (Clonetech), 100 µg/ml streptomycin, 100 U/ml penicillin and 2 mM L-glutamine with humidified atmosphere, and 5% CO_2_ at 37°C.

### SSX-RNA Interference

A suitable RNA interference (RNAi) oligonucleotide targeting all SSX isoforms SSX (1, 2, 3, 4, 4B, 5, 7, 8, and 9) and the SS18/SSX1, SS18/SSX2 and SS18/SSX4 fusion transcripts expressed in synovial sarcomas was identified by aligning the mRNA sequences of exon 5, exon 6 and 3′UTR region of SSX1 to SSX5 using established RNAi criteria. Inserts were designed to contain a 19-bp SSX mRNA sequence separated by a 9-nucleotide non-complementary spacer, and followed by the reverse complementary 19-bp mRNA sequence (Figure 2A). For shRNA vector control, a scrambled sequence containing 3 mismatched positions was also cloned into the vectors. shRNA oligonucleotides were cloned into pSuper (for stable) and pSuperior (doxycycline inducible) vectors (Oligoengine) and screened by PCR and DNA sequencing.

For SSX silencing in the cell lines we used both shRNA vectors and also siRNA molecules. To investigate the specificity of the RNAi-SSX system and to control off-target effects The effect of siRNA-SSX used in this study was compared to commercially available shRNA vectors targeting SSX1 and SSX2 in 3 independet cell lines (DFW, U2OS and SAOS-2. This is shown in the supplementary figure 2.

For transient transfections DFW was transfected with pSuper SSX shRNA (SSXi) or control RNA vector (SSXm) together with an empty vector carrying a neomycin resistance cassette. Cells were selected after transfection in G418 containing media, and expanded in bulk prior to the assays. For generation of inducible siRNA cells, DFW cells were transfected with the tetracycline repressor expressing vector pcDNA6/TR (Invitrogen) and pSuperior vector using Lipofectamine 2000 (Invitrogen) according to manufacturer’s instructions and selected with 8 µg/ml blasticidin and 1 µg/ml puromycin. Cell clones were selected based on their efficiency in silencing SSX expression following the addition of doxycycline at a concentration of 10 µg/ml as evaluated by western blot.

For gene transcription studies, wild type DFW or Saos-2 were transfected with siRNA-SSX or control siRNA molecules (Ambion). Efficient silencing of SSX was confirmed in the transfected clones at 8 and 24 hrs prior to RNA isolation and Q-RT-PCR arrays.

For simplification SSX silenced cells are referred to as SSX- and control cells as SSX+.

### Cell Cycle Synchronization and Analysis

Cells were synchronized in G1/S phase by double thymidine block.

Briefly; cells were plated to 50% confluence and treated with 2 mM thymidine for 12 hr, tripzinized, replated and released into normal medium for 8 hr, and blocked again in medium containing 2 mM thymidine for 12 hrs. For conditional silencing of SSX in syncronized DFW cells, doxycycline (10 µg/ml) was added to cells 2 hrs before releasing cells into normal medium and cell samples were then collected at indicated time points and analyzed by Propidium Iodide staining, or 5′-Bromo-2′-Deoxy-Uridine (BrdU) (Roche Diagnostics) staining and FACS analysis. The percentage of cells in each phase of the cell cycle was calculated using ModFit software.

### Antibodies, Immunoblotting and Immunoprecipitation

For SSX immunoprecipitation, cultured cells were lysed in RIPA lysis buffer (50 mM Tris-HCl pH 7.4, 150 mM NaCl, 1 mM EDTA, 0.25% Na deoxycholate, 1% NP-40) supplemented with protease inhibitors (Roche). Samples were then sonicated and centrifuged for 10 min (14000×g). 100 µg protein from the resulting supernatant was resuspended in a total volume of 1 ml with PBS containing protease inhibitors and immunoprecipitated overnight at 4°C with with 3 µg anti SSX 1–9 antibody fl188 (Santa Cruz Technologies) coupled to protein G-dynabeads (Invitrogen). The immunoprecipitates were washed once with lysis buffer and twice with PBS resuspended in loading buffer (Invitrogen), heated at 70°C for 5 minutes and resolved in 4–12% polyacrylamide gels for western blotting.

For detection of proteins by western blot, the cells were lysed *in situ* with lysis/loading buffer; sonicated and heated at 70°C and resolved in 4–12% polyacrylamide gel electrophoresis for western blotting.

The following antibodies were used: FL188 (raised in rabbit) for detection of SSX 1–9, and N18 (raised in goat) for detection of SSX1–4, 6 and 8, (Santa Cruz Biotechnologies), and an in house produced polyclonal antibody against a SSX peptide sequence (SSX PAb); antibodies against cyclin-E (HE-12, Santa Cruz); β-catenin (BD Biosciences Pharmingen), Erk, pErk, Akt, pAkt (Cell Signalling). For chemiluminiscence detection we used horseradish peroxidase-coupled conjugates and enhanced chemiluminescence substrate detection (ECL and Super Signal West Femto, Pierce).

### Cell Viability Assay

Cell viability was evaluated counting the number of alive and dead cells using trypan blue dye exclusion cell counts in triplicate. In brief 50,000 were seeded in 12 well plates and treated with 10 µg/ml doxycycline. Cells were trypsinized and stained with trypan blue at indicated time points. Each condition was run in triplicate and all experiments were repeated at least three times.

Cell proliferation was determined in real time using the xCELLigence cell analyzer (Asea BioSciences) that measures the electrical impedance of adherent cells and is expressed in arbitrary units (cell index). Cell index is dependent on cell adherence, morphology and proliferation.

### Colony Formation Assay

1×10^3^ DFW SSXi cells were plated in 3 cm cell culture plates (in triplicates). Cells were grown in the presence or absence of 10 µg/ml doxycycline for a period of 8 days. At end point of the experiment, the colonies were stained with Giemsa solution (Merck) and the number of colonies formed was counted.

### TCF/Lef Luciferase Reporter Assay

The effect of SSX on the transcription of TCF/Lef regulated genes was investigated by using a TCF/Lef luciferase reporter assays (Cignal™, SABioscience, Qiagen).

DFW and Saos-2 cells were transfected in triplicates with either siRNA-SSX or mismatched control siRNA, and a TCF (firefly-LUC) with a constitutively expressed (renilla-LUC) reporter (transfection control) was added to all transfections. The activity of the TCF reporter (firefly luciferase) was normalized to that of renilla luciferase, 48 hrs after transfection and the TCF reporter activity in SSX silenced cells was was calculated in relation to SSX expressing control cells (value = 1).

### PCR Arrays

Total RNA was isolated from SSX expressing or SSX silenced DFW or Saos-2 cells. The quality and quantity of RNA was evaluated using the Agilent Bioanalyzer prior to generation of cDNA. The RT^2^ Profiler™ PCR Array (SAB Biosciences, Qiagen) was used to profile the expression of 84 EMT genes including several β-catenin targets. The data was analyzed using a web-based PCR array data analysis (SAB biosciences, Quiagen).

The resulting data was also confirmed by Q-RT-PCR using Taq-Man probes.

### Tumorigenicity Assays in SCID Mice

Xenograft studies were performed either with inoculated DFW cells steadily expressing shRNA molecules targeting SSX (Fig. 6A) or DFW cells transfected with a doxycycline inducible shRNA vector (Fig. 6B). In both the stable and the inducible model, cells transfected with a control shRNA vector were used as SSX+ controls. BALB/c SCID mice (∼8 weeks old, 6 per group) were inoculated subcutaneously with 5×106 cells resuspended in 100 µl matrigel (BDBiosciences, San Jose, CA, USA) in the right flank and tumors were allowed to grow. For the conditional silencing of SSX, a 21day pellet mantaining 10 µg/ml/day blod concentration of doxycycline or a placebo pellet (Innovative Research of America) was introduced into the upper back of the mice once the tumors were established. Animals were sacrificed once tumors exceed 1 cm^3^ and tumors were excised for further histological investigation and for protein and RNA sample preparation. Tumor volumes were calculated using the equation *(l×w^2^)/2*, where *l* and *w* represent the largest and smallest dimensions at each In brief, 10^7^ cells were injected subcutaneously into the left flanks (SSX+ control cells) and right flanks (SSX silenced cells) of SCID mice respectively and the tumor growth was followed for 2 or 3 weeks.

### Immunohistochemistry of Paraffin Tumor Tissue Sections

Tumor tissue was excised and fixed in formalin and embedded in paraffin. The tumors were cut in 4 µm sections and the sections were placed on object slides. Paraffin was cleared with xylene and the tumor sections were re-hydrated and stained with Mayers hematoxylin and eosin. Protein expression was analyzed in the sections using the AvidinBiotin Complex method (VECTASTAIN Elite ABC Kit, Vector Laboratories). Heat-induced epitope retrieval with citrate buffer (pH 6) was performed for these sections via microwave oven at 98°C for 20 minutes. Endogenous peroxidase activity was blocked with H_2_O_2_. Peroxidase activity was developed with 3-3-diaminobenzidine (Sigma-Aldrich) to obtain a brown end product. Representative sections were stained with the following antibodies: Ki-67 (1∶75, DAKO, Denmark) and β-catenin (1∶200, BD Bioscience).

## Supporting Information

Figure S1 **RT-PCR for detection of SSX1 to SSX9.** Sequence of the PCR primers, annealing temperature and PCR fragment size. Below: ethidium bromide stained agarose gel showing amplified SSX fragments in the osteosarcoma cell lines: Saos-2, IOR-OS10 and IOR-OS9.
(TIF)

Figure S2 **SSX knockdown with independent siRNA systems.** Alignement of the SSX1 to SSX5 mRNA and siRNA sequence used in the generation of pSUPER and pSuperior shRNA vectors. A) Specificity and efficiency of the SSX-shRNA knockdown tested in the melanoma cell line DFW and in the synovial sarcoma cell line Cme-1, 24 hrs following the conditional silencing of SSX with doxycycline. C) Comparison of 3 independent RNAi-SSX systems on the proliferation of 3 tumor cell lines evaluated in real time using the xCELLigence analyzer. Cell index is quantitative measure of cell number present in a well and is determined by the change in electrical impedance., as the result of cell adhesion, morphology and proliferation. The efficiency of SSX knockdown is shown by western blot under each proliferation curve.
(TIF)

Figure S3 **Immunoprecipitation of β-catenin using SSX antibodies and the reverse experiment: Immunoprecipitation of SSX using β-catenin antibodies from DFW cell extracts.** DFW cells were blocked in G0 by serum starvation and released from the block in serum containing medium, and protein extracts were collected from cells at the indicated times. SSX or β-catenin was immunoprecipitated from 100 µg of protein using the rabbit antibody (fl188, SC technologies) that recognizes SSX1 to SSX9 isoforms or with a rabbit anti β-catenin antibody (Cell Signalling). Western blotting was performed with a goat anti SSX (N18, SCtechnologies) or a mouse anti β-catenin (Cell Signalling). As control, 100 µg protein from G0 blocked cells were immunoprecipitated with rabbit serum. SSX was detected as 2 protein bands of aproximately molecular size above 20 kD and as 2 bands of size below 19 kD.
(TIF)

Table S1 **Transcriptional changes associated with SSX knock-down.** Determined by Q-RT-PCR arrays as explained in material and methods. nd: not detected *Fold-Regulation represents fold-change results in a biologically meaningful way. Fold-change values greater than one indicate a positive- or an up-regulation.
(TIF)
