# Supplementary figures and images for: Oncogenic Functions of the Cancer-Testis Antigen SSX on the Proliferation, Survival, and Signaling Pathways of Cancer Cells
Source: PLoS One. 2014 Apr 30;9(4):e95136. doi: 10.1371/journal.pone.0095136 (PMC4005730; doi:10.1371/journal.pone.0095136)

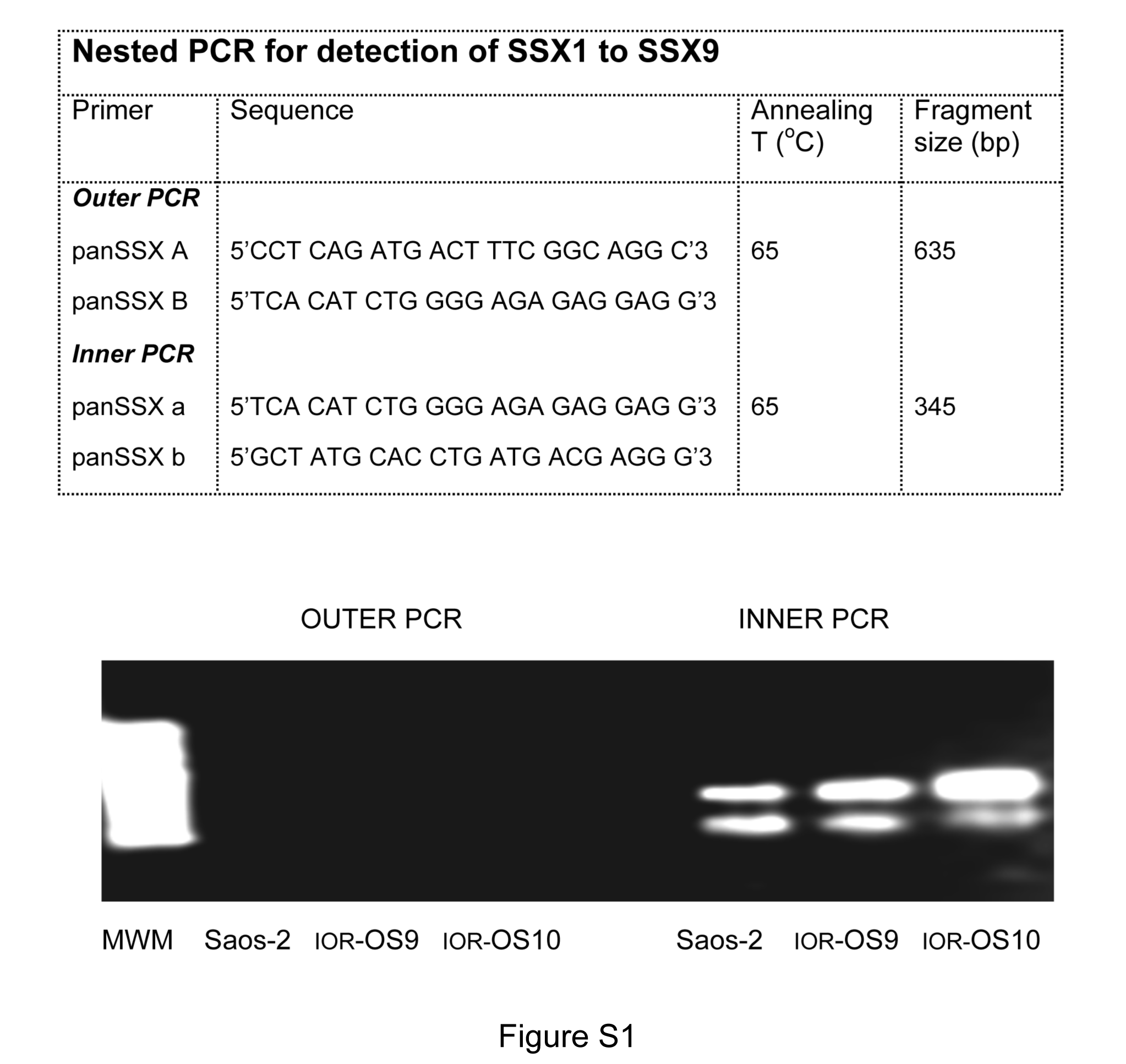

Supplement: Figure S1 — RT-PCR for detection of SSX1 to SSX9. Sequence of the PCR primers, annealing temperature and PCR fragment size. Below: ethidium bromide stained agarose gel showing amplified SSX fragments in the osteosarcoma cell lines: Saos-2, IOR-OS10 and IOR-OS9. (TIF) [file pone.0095136.s001.tif]

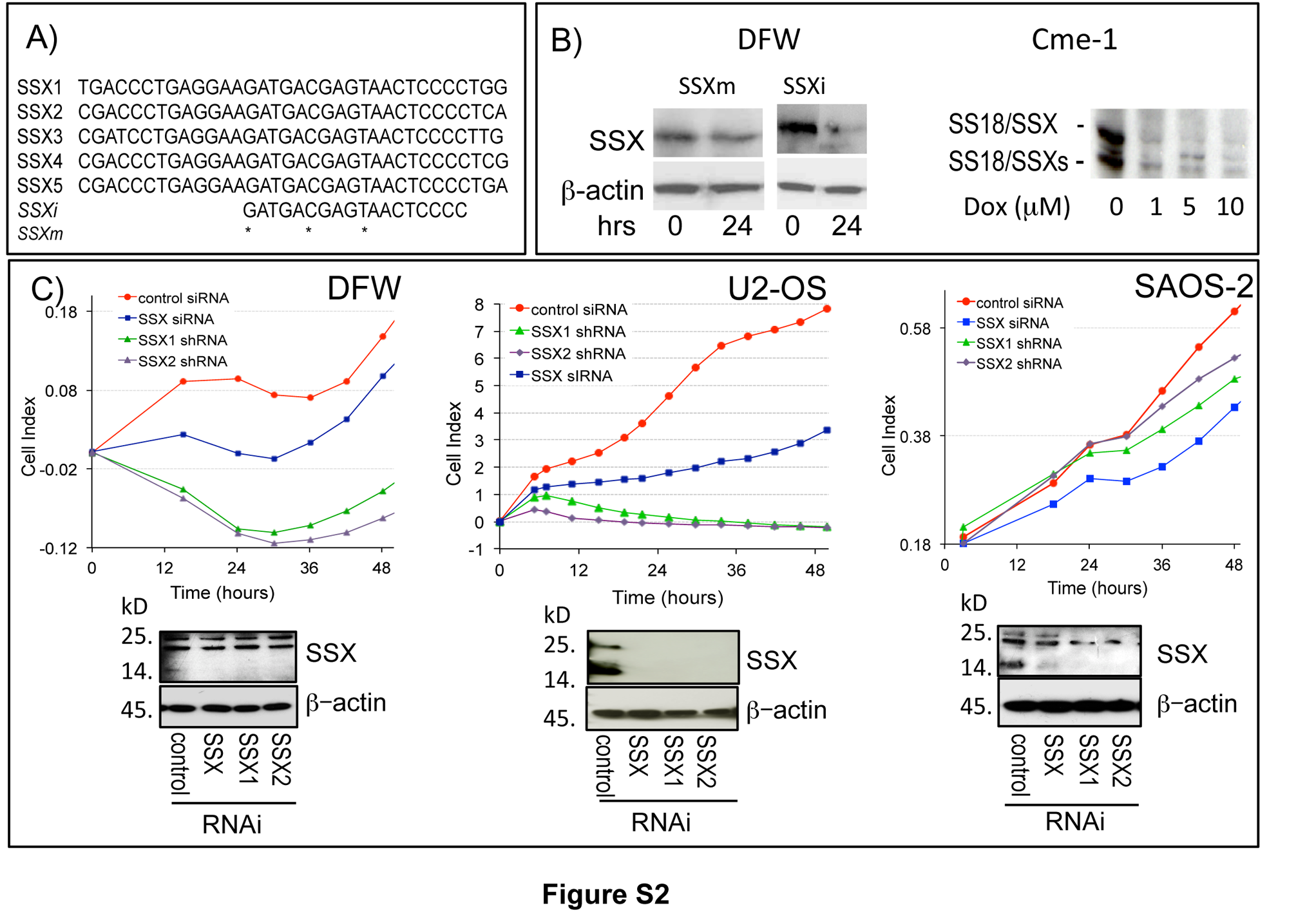

Supplement: Figure S2 — SSX knockdown with independent siRNA systems. Alignement of the SSX1 to SSX5 mRNA and siRNA sequence used in the generation of pSUPER and pSuperior shRNA vectors. A) Specificity and efficiency of the SSX-shRNA knockdown tested in the melanoma cell line DFW and in the synovial sarcoma cell line Cme-1, 24 hrs following the conditional silencing of SSX with doxycycline. C) Comparison of 3 independent RNAi-SSX systems on the proliferation of 3 tumor cell lines evaluated in real time using the xCELLigence analyzer. Cell index is quantitative measure of cell number present in a well and is determined by the change in electrical impedance., as the result of cell adhesion, morphology and proliferation. The efficiency of SSX knockdown is shown by western blot under each proliferation curve. (TIF) [file pone.0095136.s002.tif]

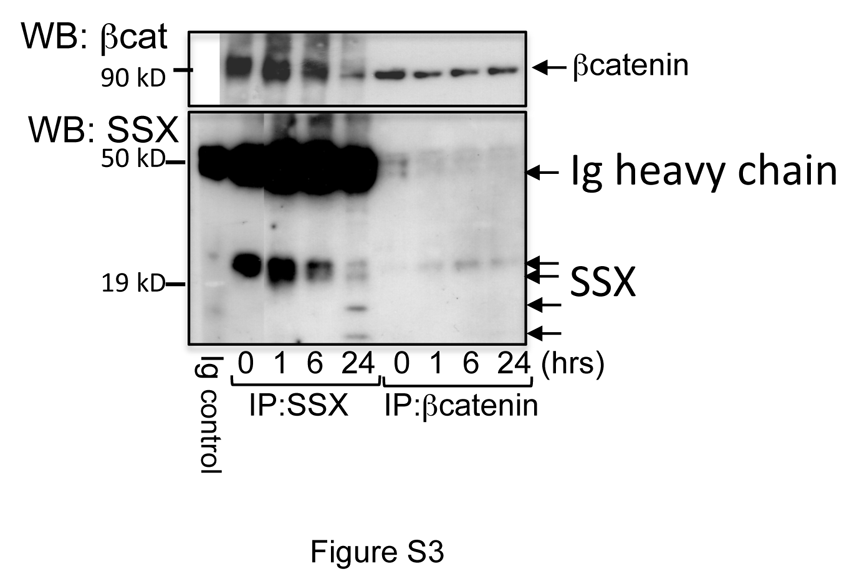

Supplement: Figure S3 — Immunoprecipitation of β-catenin using SSX antibodies and the reverse experiment: Immunoprecipitation of SSX using β-catenin antibodies from DFW cell extracts. DFW cells were blocked in G0 by serum starvation and released from the block in serum containing medium, and protein extracts were collected from cells at the indicated times. SSX or β-catenin was immunoprecipitated from 100 µg of protein using the rabbit antibody (fl188, SC technologies) that recognizes SSX1 to SSX9 isoforms or with a rabbit anti β-catenin antibody (Cell Signalling). Western blotting was performed with a goat anti SSX (N18, SCtechnologies) or a mouse anti β-catenin (Cell Signalling). As control, 100 µg protein from G0 blocked cells were immunoprecipitated with rabbit serum. SSX was detected as 2 protein bands of aproximately molecular size above 20 kD and as 2 bands of size below 19 kD. (TIF) [file pone.0095136.s003.tif]

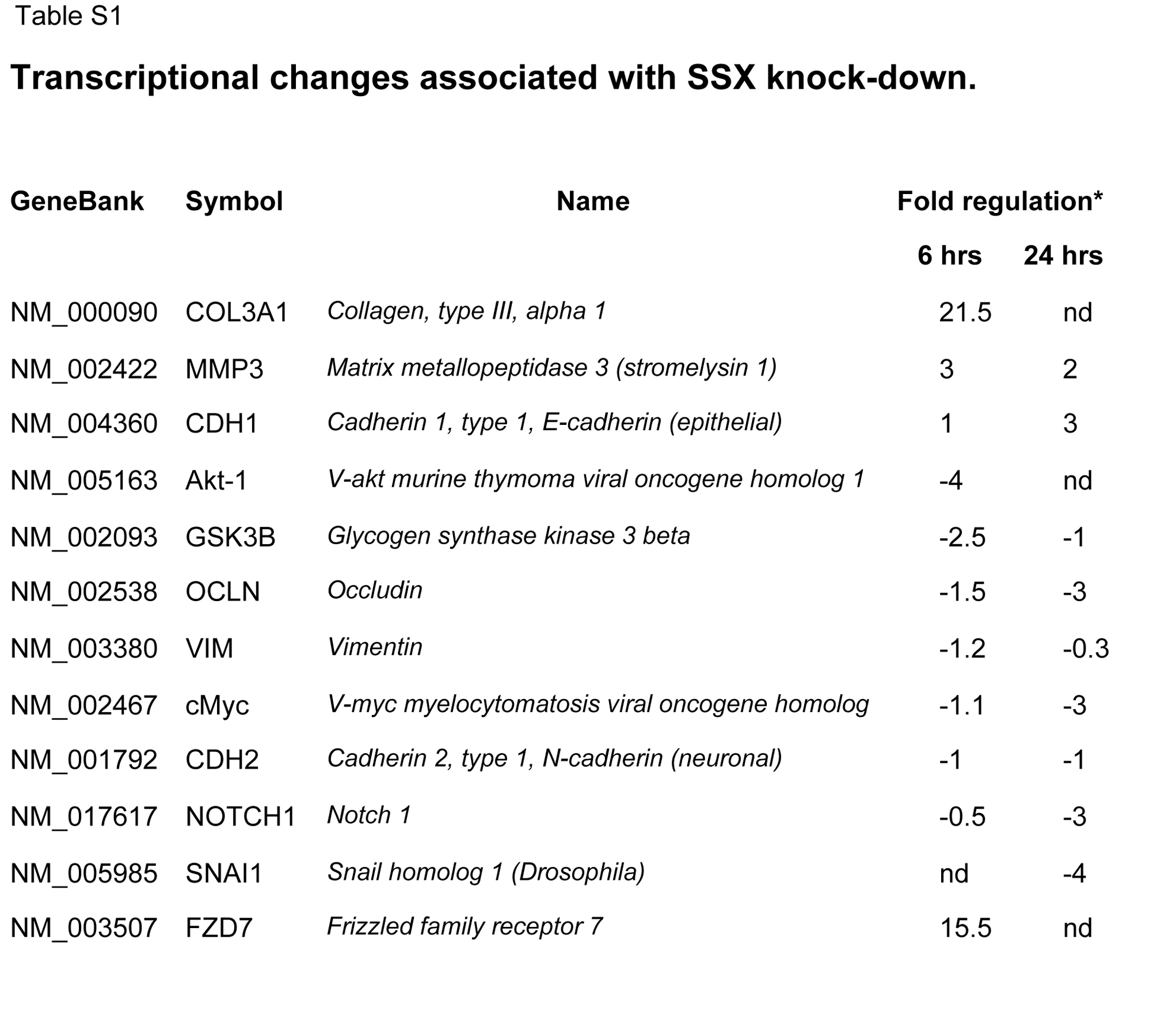

Supplement: Table S1 — Transcriptional changes associated with SSX knock-down. Determined by Q-RT-PCR arrays as explained in material and methods. nd: not detected *Fold-Regulation represents fold-change results in a biologically meaningful way. Fold-change values greater than one indicate a positive- or an up-regulation. (TIF) [file pone.0095136.s004.tif]
